# Rapid and Sensitive Fluorescent RT-RAA Assay for the Detection of a Panel of Six Respiratory Viruses

**DOI:** 10.3390/diagnostics16010009

**Published:** 2025-12-19

**Authors:** Xudong Guo, Dongli Gao, Yi Yang, Wanying Liu, Hongbo Liu, Rongtao Zhao, Hongbin Song

**Affiliations:** 1 Chinese PLA Center for Disease Control and Prevention, Beijing 100071, China; 2 Jiangyin Center for Disease Control and Prevention, Jiangyin 214400, China

**Keywords:** isothermal amplification, recombinase-aided amplification (RAA), respiratory virus, point-of-care testing, portable device

## Abstract

**Background**: Rapid pathogen detection is crucial for the timely containment of outbreaks, particularly for respiratory infectious diseases which are highly transmissible and possess high epidemic potential. **Methods**: We developed a sensitive reverse transcription recombinase-aided amplification (RT-RAA) assay for the rapid detection of six common respiratory viruses: respiratory syncytial virus type A (RSV A), influenza A virus (Flu A), influenza B virus (Flu B), human parainfluenza virus (HPIV), SARS-CoV-2 and adenovirus (ADV). The assay employs a single, standardized protocol for the on-demand detection of any one of the six targets. Its performance was validated using nucleic acid standards and clinical pharyngeal swab specimens. **Results**: The assay enables rapid detection within 20 min at 39 °C using a portable, self-powered device. It demonstrated high sensitivity, with detection limits below 10^3^ copies/mL for all targets and as low as 10^1^ copies/mL for ADV. Cross-reactivity testing with 21 other pathogens confirmed excellent specificity. Validation with 85 clinical samples showed 100% concordance with RT-PCR, while offering significantly faster results and enhanced portability compared to RT-PCR. **Conclusions**: This sensitive, specific, and user-friendly RT-RAA assay provides a robust tool for rapid detection of respiratory viruses, particularly suitable for deployment in resource-limited settings and point-of-care testing during outbreaks.

## 1. Introduction

Acute respiratory infections caused by viruses such as influenza, respiratory syncytial virus (RSV), and SARS-CoV-2 pose a persistent global health threat due to their high transmissibility and potential severity [1,2,3]. The non-specific nature of early symptoms and the risk of asymptomatic transmission underscore the critical need for rapid, accurate, and accessible diagnostic tools at the point of need to enable timely intervention and effective outbreak control [4,5].

Nucleic acid amplification tests (NAATs), particularly polymerase chain reaction (PCR) as the gold standard, are foundational to modern pathogen detection due to their exceptional sensitivity and specificity [6,7,8]. However, their fundamental reliance on precise thermal cycling necessitates sophisticated instrumentation, stable laboratory infrastructure, and extended processing times, which severely limit their utility in resource-limited, community, or point-of-care (POC) settings [9,10]. Immunological assays such as enzyme-linked immunosorbent assay (ELISA) and colloidal gold assay are rapid but suffer from relatively low sensitivity, leading to an increased risk of false-negative results [11,12]. This dichotomy creates a pressing diagnostic gap where both high accuracy and field-deployability are paramount.

Isothermal amplification technologies have emerged as promising alternatives to PCR for decentralized testing, as they amplify nucleic acids at a constant temperature, eliminating the need for thermal cyclers [13,14,15,16]. A comparative analysis of major isothermal amplification methods against PCR reveals a critical trade-off [17,18,19] (Table S1). The requirement for precise thermal cycling imposes inherent constraints on the deployment of PCR in point-of-care or resource-limited environments. These limitations primarily manifest in three aspects: a prolonged turnaround time (typically 60–120 min), a dependency on sophisticated and costly thermocycling instrumentation, and a reliance on stable electrical power and controlled laboratory conditions. Collectively, these factors hinder the utility of conventional PCR for rapid, decentralized testing where simplicity, speed, and operational independence are paramount. In contrast, isothermal amplification techniques circumvent the need for thermal cycling by operating at a constant temperature. This fundamental difference enables a significant reduction in instrumental complexity and facilitates faster reaction kinetics.

However, techniques like Loop-Mediated Isothermal Amplification (LAMP) offer high sensitivity, they require complex primer design (4–6 primers) and high operating temperatures (65 °C) [20,21,22,23,24]. Nucleic Acid Sequence-Based Amplification (NASBA), while sensitive for RNA, involves multiple enzymes and longer reaction times (90 min) [25,26]. In contrast, recombinase-aided amplification (RAA) and its derivative reverse transcription RAA (RT-RAA) operate efficiently at a lower, flexible temperature range (37–42 °C) [27], use simple primer pairs akin to PCR, and deliver results within 20 min [28,29]. This unique combination of operational simplicity, rapid kinetics, and minimal equipment requirements positions RAA as the most promising isothermal platform for developing true point-of-care (POC) molecular diagnostics [30].

The application of RT-RAA in respiratory virus detection has seen significant progress. It has been successfully validated for individual pathogens such as SARS-CoV-2 [28,31], influenza virus [32], and RSV [33], demonstrating excellent clinical agreement with RT-PCR. Furthermore, its potential for syndromic testing is being explored, with recent advances in multiplex RT-RAA assays capable of simultaneously detecting two or three pathogens in a single tube [34]. The portability of RT-RAA has also been proven in real-world point-of-care testing scenarios during the COVID-19 pandemic [35]. However, a significant translation gap remains between its technical promise and practical, field-ready application. Existing platforms are often limited to single-plex detection or fixed multiplex panels, lack integrated portable and self-powered instrumentation, and do not offer the flexible throughput needed for efficient testing in diverse field scenarios.

To directly address these specific limitations, this study developed and validated an integrated, field-deployable diagnostic platform. We established a versatile “one-protocol-for-all” fluorescent RT-RAA assay for the simultaneous on-demand detection of six major respiratory viruses (RSV A, influenza A/B, human parainfluenza virus, SARS-CoV-2, and adenovirus). Crucially, this assay was coupled with a custom-built, portable, and battery-powered detection device featuring a 16-channel capacity. This integrated design ensures full operational autonomy, enables flexible batch processing of multiple samples or targets, and eliminates dependency on external power and benchtop instruments—key requirements for deployment in clinics, field stations, or during outbreak investigations. Through systematic primer screening, rigorous analytical validation, and clinical testing, our platform bridges the critical gap from a laboratory technique to a practical, robust, and user-friendly solution for rapid, multiplex-capable respiratory pathogen detection at the point of care.

## 2. Materials and Methods

### 2.1. Overall Workflow

**Figure 1:**
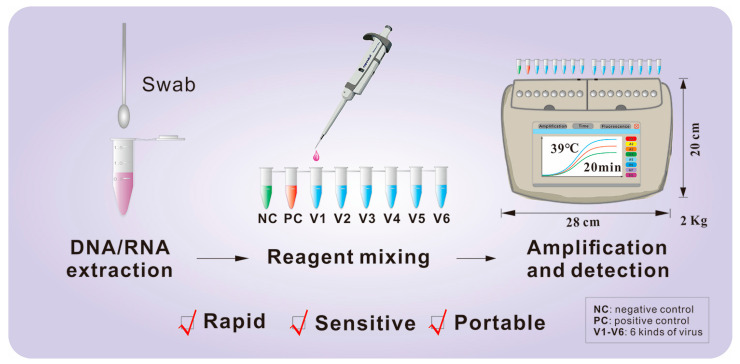
Schematic workflow of the fluorescent RT-RAA assay. The procedure comprises three main stages: (1) viral nucleic acid (DNA/RNA) extraction from clinical swab samples; (2) preparation and mixing of the RT-RAA master mix with the extracted template in a single tube; and (3) isothermal amplification and real-time fluorescence detection at 39 °C for 20 min using a portable device. Abbreviations: NC, negative control; PC, positive control; V1–V6, the six target respiratory viruses.

A schematic overview of the complete fluorescent RT-RAA detection process is provided in Figure 1. The assay involves three core steps: (i) viral nucleic acid extraction, (ii) master mix preparation and mixing, and (iii) isothermal amplification with real-time fluorescence detection, as detailed in the following subsections.

### 2.2. Materials

Nucleic acid standards for RSV A, HPIV, Flu A, Flu B, and SARS-CoV-2 were sourced from the Shanghai Institute of Measurement and Testing Technology, with their detailed sequence information provided in Table S2. Adenovirus (ADV) was propagated in our laboratory via chicken embryo culture. For clinical validation, a total of 85 archived clinical pharyngeal swab samples were used, comprising 71 positive and 14 negative specimens for the target viruses. Additionally, nucleic acid samples from 21 non-target pathogens were included for specificity testing. Viral nucleic acids were extracted using commercial kits from Tiangen Biochemical Technology (Beijing, China). All primers and probes were custom-synthesized by Sangon Biotech (Shanghai, China). A commercial RT-PCR kit (Shenzhen Aodong Inspection and Testing Technology Co., Shenzhen, China) served as the reference method for comparative analysis. A complete list of all reagents and reference materials is available in Table S3.

### 2.3. Instruments and Equipment

The following instruments were used: a Qitian RAA-B6100 mixer (Jiangsu Qitian Genetic Biotechnology Co., Ltd., Wuxi, China) for reagent vortexing and mixing; a Genchek fluorescent detector (Hangzhou Zhongce Bio-Sci&Tech Co. Ltd., Hangzhou, China; refer to Figure S1 for a photograph and Table S4 for specifications) for nucleic acid amplification; and a CFX96 Touch real-time PCR system (Bio-Rad Laboratories, Inc., Hercules, CA, USA) for reference RT-PCR assays. A complete list of Instruments, including their respective vendor/brand, catalog number/model, and country of origin, is provided in Table S3.

### 2.4. Design of Primers and Probes

The conserved genomic regions for each of the six target viruses were first identified as the basis for primers and probes design. Corresponding sequences were retrieved from the NCBI database (minimum of 20 sequences per virus) and aligned using DNAMAN version 6.0 (Lynnon Biosoft), with the resulting conserved sequences provided in Table S5.

An exo-probe was designed for each virus, targeting its respective conserved region with a length of 46–52 nucleotides. Each probe was modified at the 3′ end with a C3 Spacer, and a tetrahydrofuran (THF) residue was incorporated in the middle as the cleavage site for the exonuclease (exo). A FAM fluorophore and a BHQ1 quencher were conjugated to the two thymine bases immediately flanking the THF site. The 5′ end of the probe was positioned at least 30 nucleotides upstream of the THF, while the 3′ end was at least 15 nucleotides downstream.

Primer sets were then designed to flank the probe binding sites using the Primer-BLAST online tool [36], with the following parameters: amplicon size of 120–350 bp, primer length of 28–35 nucleotides, GC content of 20–70%, maximum self-complementarity of 4.0, and a melting temperature (Tm) of 57–63 °C. Multiple candidate primer pairs were generated for each virus to facilitate subsequent screening.

### 2.5. Primer Screening

Based on our team′s established primer development strategy, a two-round screening approach was employed to identify optimal primer-probe combinations.

Primary Optimization (In silico Design and Initial Experimental Screening): Candidate primer pairs were designed using the Primer-BLAST online tool. Initial selection was based on design scores, with emphasis on optimal self-complementarity and minimal 3′-end complementarity to ensure primer specificity. The top-scoring candidates were then evaluated experimentally.

Binding Site Adjustment: The best-performing primer sets from the primary optimization were subjected to a second round of refinement. To fine-tune amplification efficiency, the binding sites of these primers were systematically shifted upstream or downstream along the same conserved genomic region while maintaining their original length. This process generated new primer variants for comparative evaluation.

Final Selection via Analytical Validation: The most promising 1–2 primer-probe sets emerging from the secondary optimization for each virus underwent comprehensive analytical validation. The final, optimal set for each target was definitively selected based on superior performance in rigorous quantitative tests of analytical sensitivity (determination of the lowest limit of detection) and analytical specificity (evaluation against a full panel of non-target pathogens).

Screening and Evaluation Criteria: All experimental screening and optimization reactions were conducted using a template concentration of 10^5^ copies/mL. Real-time fluorescence curves were analyzed with a fixed fluorescence threshold set at 5000 RFU. Candidate sets were assessed and prioritized based on three key kinetic parameters: (i) a short time to positive (Tp), defined as the time point at which the amplification curve crossed the 5000 RFU threshold (typically targeting Tp < 10 min); (ii) a high endpoint fluorescence intensity; and (iii) the absence of any signal in non-template negative controls.

### 2.6. RT-RAA Fluorescence Assay

The RT-RAA reaction was assembled according to the manufacturer′s instructions. A master mix was prepared containing nuclease-free water, Buffer A, pre-primer, forward primer, and reverse primer, with specific volumes listed in Table S6. This mixture was transferred to a reaction tube pre-loaded with lyophilized reaction pellet. The RNA/DNA template was then added. Subsequently, 2.5 μL of Buffer B was applied to the interior of the tube cap, and the tube was sealed securely. The sealed tube was first incubated in a Qitian RAA-B6100 mixer for a 4-min pretreatment step, after which it was immediately transferred to a Genchek fluorescence detector for amplification at 39 °C for 20 min.

### 2.7. Performance Evaluation

#### 2.7.1. Analytical Sensitivity Evaluation

The analytical sensitivity of each assay was determined by testing tenfold serial dilutions of the corresponding nucleic acid standard, ranging from 10^5^ to 10^0^ copies/mL. Nuclease-free water was included as a negative control. The limit of detection (LoD) was defined as the lowest concentration at which the target was consistently amplified, with all LoD experiments performed in at least three independent replicates.

#### 2.7.2. Analytical Specificity Evaluation

The specificity of each primer-probe set was evaluated against a panel of 21 non-target pathogens to assess potential cross-reactivity. The panel included: RSV A, RSV B, HPIV, H1N1, Influenza B virus, SARS-CoV-2, Adenovirus, Rhinovirus (types A, B, and C), H7N9, human coronaviruses (OC43, 229E, NL63), HMPV, MERS-CoV, and the bacterial species *Staphylococcus aureus*, *Chlamydia pneumoniae*, *Mycoplasma pneumoniae*, and *Klebsiella pneumoniae.* Nucleic acid templates extracted from all non-target pathogens were tested at a high concentration of 10^5^ copies/mL.

#### 2.7.3. Clinical Validation

A total of 85 archived clinical pharyngeal swab samples were used to validate the clinical performance of the RT-RAA assay against a commercial RT-PCR kit (reference method). The sample set comprised 71 samples previously confirmed positive for a single target virus and 14 samples negative for all six target viruses. The positive samples included: 11 Respiratory Syncytial Virus type A (RSV A), 6 Influenza A virus (Flu A), 30 Influenza B virus (Flu B), 5 Human Parainfluenza Virus (HPIV), 11 SARS-CoV-2, and 8 Adenovirus (ADV).

Viral RNA/DNA was extracted from all samples. For clinical validation, each of the six virus-specific RT-RAA assays was tested against a focused panel: To establish clinical sensitivity, each assay was tested against the subset of samples positive for its corresponding target virus. To establish clinical specificity within a target-negative clinical matrix, each assay was tested against the 14 samples negative for all six target viruses.

All samples were tested in parallel using both the RT-RAA assay and the reference RT-PCR method. The RT-PCR was performed on a Bio-Rad CFX96 Touch system in a 25 µL reaction volume, strictly following the manufacturer′s instructions. The concordance between the two methods was statistically analyzed using SPSS software (version 24.0).

## 3. Results and Discussion

### 3.1. Screening of Primers and Probes

**Figure 2:**
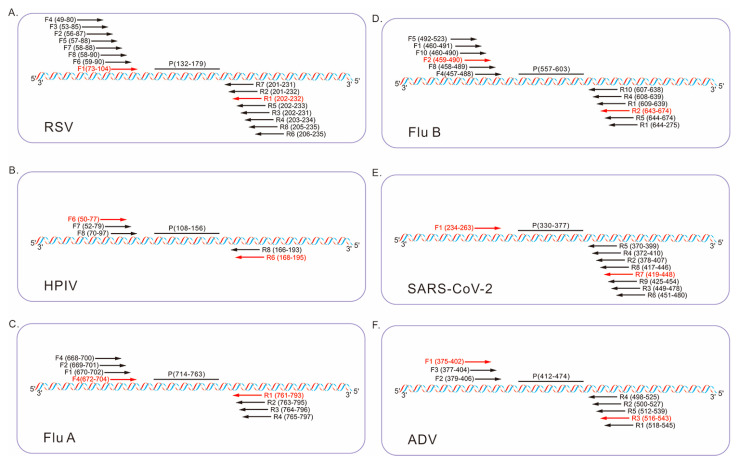
Schematic diagram illustrating the relative positions of the fluorescent probes (P) and candidate primers (F, forward; R, reverse) within the conserved regions of the six respiratory viruses. The optimal primer pairs are highlighted in red. The numbers in parentheses represent relative positions within the following reference sequences: (**A**) RSV A (GenBank: KX655697.1, region 4610–5503), (**B**) HPIV (GenBank: MF554715.1, region 82–1656), (**C**) Flu A (GenBank: NC_002018.1, region 1–1014), (**D**) Flu B (GenBank: CY018656.1, region 45–1727), (**E**) SARS-CoV-2 (GenBank: OQ253304.1, region 28214–29464), (**F**) ADV (GenBank: MW816018.1, region 18385–21189).

The relative positions of primers and probes from the two-round optimization are illustrated in Figure 2, where the specific binding sites of each probe are annotated as P (132–179) for RSV, P (108–156) for HPIV, P (714–763) for Flu A, P (557–603) for Flu B, P (330–377) for SARS-CoV-2, and P (412–474) for ADV. The ideal primer pairs were selected based on real-time fluorescence curves (Figure S2), with optimal sets demonstrating strong endpoint fluorescence intensity, short time to positive, and absence of non-specific amplification in negative controls. The sequences of the final selected primer and probe sets are summarized in Table 1.

Based on our experience, the performance of a diagnostic assay is highly dependent on the rigor of its initial design and screening phase. The meticulous optimization described here—which prioritizes amplification kinetics, specificity, and signal strength through screening a broad panel of candidate primers—is expected to translate into superior detection performance, as validated in the subsequent sections.

### 3.2. Analytical Sensitivity Results

**Figure 3:**
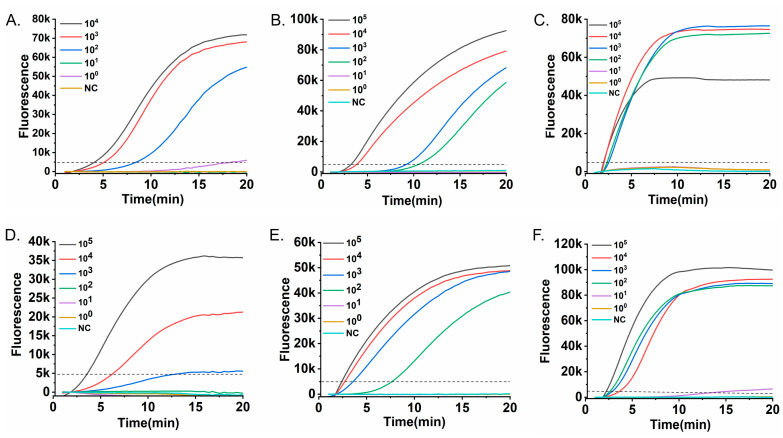
Analytical sensitivity of the RT-RAA assays. Serial tenfold dilutions of nucleic acid standards were tested to determine the detection limit for each virus. Fluorescence-time curves are shown for (**A**) RSV A RNA (10^4^–10^0^ copies/mL), (**B**) HPIV RNA (10^5^–10^0^ copies/mL), (**C**) Flu A RNA (10^5^–10^0^ copies/mL), (**D**) Flu B RNA (10^5^–10^0^ copies/mL), (**E**) SARS-CoV-2 RNA (10^5^–10^0^ copies/mL), and (**F**) ADV DNA (10^5^–10^0^ copies/mL). The dashed horizontal line indicates the fluorescence threshold (5000 RFU). The limit of detection for each virus, confirmed in three independent replicates, was defined as the lowest concentration at which all replicates consistently exceeded the threshold within the 20-min assay.

The analytical sensitivity of the RT-RAA assays for the six respiratory viruses was evaluated using tenfold serial dilutions of the corresponding RNA/DNA standards. Real-time fluorescence curves were acquired with the portable Genchek detector (Figure 3). The limits of detection (LoD) were as follows: 10^2^ copies/mL for RSV A (Figure 3A), 10^2^ copies/mL for HPIV (Figure 3B), 10^2^ copies/mL for Flu A (Figure 3C), 10^3^ copies/mL for Flu B (Figure 3D), 10^2^ copies/mL for SARS-CoV-2 (Figure 3E), and 10^2^ copies/mL for ADV (Figure 3F).

These detection limits are comparable to those of conventional RT-PCR, yet are achieved in ≤20 min without the need for thermal cycling, highlighting a key advantage in speed and operational simplicity for time-sensitive applications. This simplicity is further underscored by the use of a portable, all-in-one detector that integrates heating, optics, and analysis, eliminating the need for complex instrument setup and significantly streamlining the workflow.

### 3.3. Analytical Specificity Results

**Figure 4:**
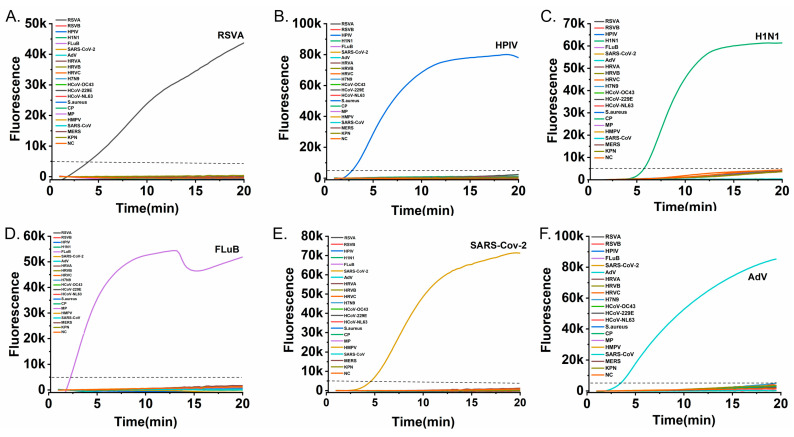
Specificity testing of the six RT-RAA assays against a panel of non-target pathogens. Each subfigure presents amplification curves for a specific target alongside non-target pathogens: (**A**) RSV A, (**B**) HPIV, (**C**) Flu A, (**D**) Flu B, (**E**) SARS-CoV-2, and (**F**) ADV. The dashed horizontal line indicates the fluorescence threshold (5000 RFU). Amplification curves rising above the threshold were observed only for their respective target viruses (colored curves while all non-target pathogen curves remained below the threshold, confirming no cross-reactiv and the high specificity of each assay).

The specificity of the six RT-RAA assays was evaluated against a panel of 21 non-target pathogens, comprising other respiratory viruses and clinically relevant bacteria (RSV A, RSV B, HPIV, Flu A, Flu B, SARS-CoV-2, ADV, rhinovirus types A–C, H7N9, HCoV-OC43, HCoV-229E, HCoV-NL63, HMPV, MERS-CoV, *Staphylococcus aureus*, *Chlamydia pneumoniae*, *Mycoplasma pneumoniae*, and *Klebsiella pneumoniae*). As demonstrated in Figure 4, amplification was observed only for the respective target pathogens, with no cross-reactivity detected for any non-target nucleic acids. These results confirm the high specificity of each primer-probe set.

This high specificity, critical for accurate diagnosis, is on par with gold-standard PCR and superior to many rapid antigen tests. It underscores the robustness of our primer-probe designs in complex pathogen backgrounds. This achievement is particularly notable given the isothermal nature of RAA, which operates at a constant lower temperature.

### 3.4. Clinical Validation Results

The clinical performance of each RT-RAA assay was evaluated against the reference RT-PCR method using 71 pre-characterized positive samples (11 RSV A, 5 HPIV, 6 Flu A, 30 Flu B, 11 SARS-CoV-2, and 8 ADV) and 14 target-negative samples.

**Figure 5:**
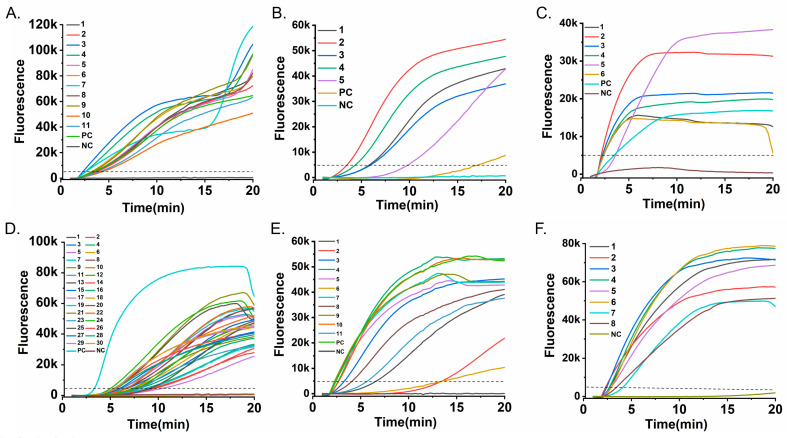
Clinical validation of the RT-RAA assay. Fluorescence-time curves obtained from testing clinical pharyngeal swab samples are shown: (**A**) 11 RSV A-positive samples; (**B**) 5 HPIV-positive samples; (**C**) 6 Flu A-positive samples; (**D**) 30 Flu B-positive samples; (**E**) 11 SARS-CoV-2-positive samples; and (**F**) 8 ADV-positive samples. The dashed horizontal line indicates the fluorescence threshold (5000 RFU) used to determine a positive result. All clinical positive samples generated curves that unequivocally crossed this threshold, demonstrating 100% detection agreement with the reference method.

All positive samples were correctly identified by their corresponding RT-RAA assay within 20 min, yielding a 100% positive percent agreement (PPA) with RT-PCR (Figure 5). Furthermore, no false-positive results were observed among the negative samples, demonstrating 100% negative percent agreement (NPA) for each assay (Supplementary Table S7).

Thus, the RT-RAA assay achieved complete diagnostic concordance with RT-PCR. This high level of agreement, combined with the substantially shorter turnaround time (≤20 min vs. typically >60 min for conventional RT-PCR) and the simplified, instrument-light workflow, supports the platform’s potential for reliable rapid testing in clinical and field settings. It should be noted that the current validation specifically confirmed assay performance for their corresponding target viruses; formal cross-reactivity testing against clinical samples containing high titers of non-target respiratory pathogens was not included in this study. Further evaluation of analytical specificity in diverse clinical matrices will be an important focus of future verification studies.

### 3.5. Integrated Platform Utility and Strategic Outlook

The establishment of this fluorescent RT-RAA platform demonstrates that high sensitivity, specificity, and speed can be integrated into a field-deployable format. The systematic primer screening contributed to consistent performance, while the portable design (2 kg) with built-in power supply enables operation independent of laboratory infrastructure. The 16-channel architecture offers flexible testing configurations, an improvement over many 8-channel point-of-care devices.

Unlike traditional PCR, our system eliminates complex thermal cycling and reduces dependency on stable power and specialized labs, directly addressing key bottlenecks in resource-limited settings. While current detection is single-plex, the platform’s inherent scalability supports future multiplex development for comprehensive syndromic testing.

**Figure 6:**
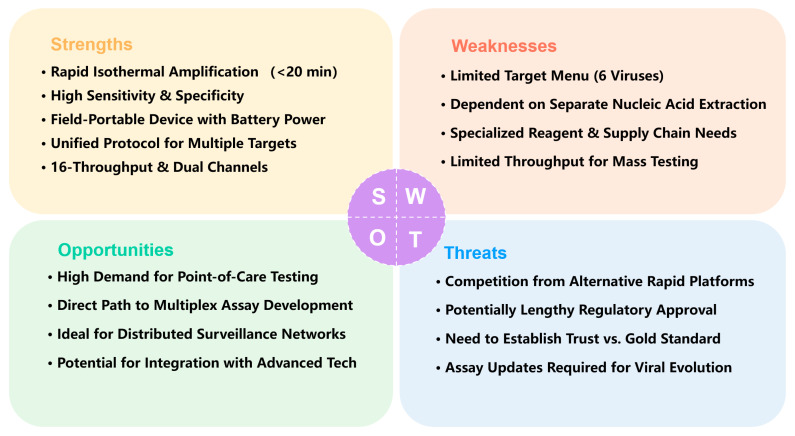
SWOT Analysis of the Portable Fluorescent RT-RAA Platform for Respiratory Virus Detection.

To strategically summarize the position and potential of this platform, a SWOT (Strengths, Weaknesses, Opportunities, Threats) analysis is provided in Figure 6.

This SWOT analysis evaluates the strategic position of the portable fluorescent RT-RAA platform for respiratory virus detection.

(a) Strengths. The core strengths of the platform lie in its speed (20 min), high sensitivity and specificity, and field portability. It employs isothermal amplification (39 °C), integrates into a portable battery-powered device (2 kg), and is suitable for point-of-care testing. With a low limit of detection (10^1^–10^3^ copies/mL) and high agreement with RT-PCR, it uses a unified protocol to detect six major respiratory viruses simultaneously, supports 16-sample throughput and dual fluorescence channels, and offers strong extensibility that provides a clear pathway for future development.
(b) Weaknesses. The platform currently has a limited target menu (only 6 viruses) and depends on separate nucleic acid extraction, hindering full workflow integration. Its reliance on specialized reagents and supply chain management may affect sustainability in resource-limited settings. The format also limits throughput for co-infection screening and mass testing. Addressing these limitations, particularly by advancing toward multiplex and extraction-free testing, is crucial for enhancing its utility and adoption.
(c) Opportunities. External opportunities are reflected in the growing demand for point-of-care testing in clinics, airports, and outbreak settings. The platform’s hardware foundation—dual channels and 16 reaction slots—provides a direct path to multiplex assay development and future integration with microfluidics or smartphone-based readouts. It is well-suited to serve as a node in distributed public health surveillance networks and has potential for integration with advanced technologies. This positions the platform to effectively seize emerging market opportunities in decentralized diagnostics.
(d) Threats. The platform faces competition from other rapid molecular and antigen tests, potentially lengthy regulatory approval processes for novel IVDs, and the need to build trust among users accustomed to PCR. Furthermore, ongoing viral evolution requires continuous assay updates, adding to maintenance and upgrade costs. Successfully navigating this competitive and evolving landscape will be key to its long-term viability.

## 4. Conclusions

The fluorescent RT-RAA assay developed in this work demonstrates significant advantages in speed (20 min), portability (2 kg, battery-powered) and sensitivity (limit of detection of 10^1^–10^3^ copies/mL) compared to conventional RT-PCR. By integrating a built-in power supply, lightweight design, and unified isothermal amplification protocol, our system provides a practical and effective solution for accurate, point-of-care detection of multiple respiratory pathogens, showing strong potential for use in resource-limited settings and field outbreak investigations.

However, the current platform has certain limitations that must be acknowledged. Its target menu remains confined to six viruses, and it still depends on separate nucleic acid extraction, which hinders full workflow integration and true sample-to-answer operation. Furthermore, the current assay format and workflow present a significant gap in meeting the demands of large-scale, high-throughput screening during pandemic outbreaks.

Looking forward, the system’s hardware foundation—including dual fluorescence channels and 16 reaction slots—offers a clear pathway for future development toward multiplex detection and integration with extraction-free protocols or microfluidic systems. Addressing these limitations will be crucial for enhancing utility and adoption. Despite competition from other rapid tests and the regulatory hurdles inherent to novel IVDs, this technology represents a meaningful step toward decentralized diagnostic capabilities. With further refinement to overcome current constraints, it holds considerable promise for strengthening respiratory disease surveillance and outbreak response strategies in diverse healthcare and public health environments.

## Figures and Tables

**Table 1:** Sequences of the final selected primers and probes for the six target respiratory viruses.

Name ^1^	Sequences (5′–3′) ^2^	Amplicon Length (bp)
RSVA-F	TTCATATCATCGTGCTTATACAAGTTAAATCT	160
RSVA-R	CTTGTATGATTGCAGTTGTTAGTGTGACTTT	
RSVA-P	TATTTTGGCAATGATAATCTCAACCTCACT[FAM-dT][THF][BHQ1-dT]AATTGCAGCCATCAT[C3-spacer]	
HPIV-F	AGAGCATCAATAAGTCTGGCGGAGGAGC	146
HPIV-R	ATCTGTATCCAGTGAGTGGGCTAAGAAA	
HPIV-P	TGTCTTCACATTAGGCCCGAGTGTGACAGA[FAM-dT][THF]A[BHQ1-dT]GCAGATAAATTATTA[C3-spacer]	
Flu A-F	TGTGTAAATGGTTCATGTTTTACTATAATGACT	122
Flu A-R	TTAGGTGCATTCAACTCTATTGATTTAGTAACC	
Flu A-P	AGTGATGGGCTGGCCTCGTACAAAATTT[FAM-dT]CA[THF]GA[BHQ1-dT]CGAAAAGGGGAAGGT[C3-spacer]	
Flu B-F	CTTTTACAAGATGGTAAGAGATGATAAAAC	216
Flu B-R	ATTAATGAAGGATCAAGTCCAACTCTTTTTAG	
Flu B-P	TGGGGAGTGATGGCTTCAGTGGATTAAA[FAM-dT]C[THF]CA[BHQ1-dT]AATGATTGGGCA[C3-spacer]	
SARS-CoV-2-F	TACGCAGAAGGGAGCAGAGGCGGCAGTCAA	215
SARS-CoV-2-R	CCTTGTTGTTGTTGGCCTTTACCAGACATT	
SARS-CoV-2-P	AAGAGCAGCATCACCGCCATTGCCAGCCAT[FAM-dT][THF][BHQ1-dT]AGCAGGAGAAGTTCC[C3-spacer]	
ADV-F	CCTATGAGCAGGCAGGTGGTTGATGAGG	169
ADV-R	CGGCAGTAGTTCCGATGAGCGGGTATGG	
ADV-P	CGTCACCTTACCATATCAACACAACAACTC[FAM-dT]G[THF]C[BHQ1-dT]TTGTAGGATACCTTG[C3-spacer]	

^1^ F, Forward primer; R, Reverse primer; P, Probe. ^2^ The probe modifications are as follows: FAM, fluorophore; BHQ1, quencher; THF, tetrahydrofuran (cleavage site); C3-Spacer, 3′ blocker.

## Data Availability

The original contributions presented in this study are included in the article/supplementary material. Further inquiries can be directed to the corresponding author.

## Supplementary Materials

The following supporting information can be downloaded at: https://www.mdpi.com/article/10.3390/diagnostics16010009/s1. Table S1. A comparison of major isothermal amplification methods with PCR highlights their distinct trade-offs. Table S2. Information on viral nucleic acid standards. Table S3. List of instruments, reagents, and reference materials. Figure S1. The portable Genchek real-time fluorescent RAA detector: photograph, physical dimensions, and key specifications. Table S4. Key technical specifications of the portable Genchek real-time fluorescent RAA detector. Table S5. Identified conserved sequences of the six target viruses. Table S6. Formulation of the RT-RAA fluorescence reaction mixture. Figure S2. Evaluation of candidate primer-probe sets for the six respiratory viruses. Table S7. Detection results of RT-RAA versus RT-qPCR in clinical pharyngeal swab samples.
