# Supplementary material for: Rapid and Sensitive Fluorescent RT-RAA Assay for the Detection of a Panel of Six Respiratory Viruses
Source: Diagnostics (Basel). 2025 Dec 19;16(1):9. doi: 10.3390/diagnostics16010009 (PMC12785408; doi:10.3390/diagnostics16010009)
Supplement: Supplementary file 1 [file diagnostics-16-00009-s001.zip › diagnostics-4027315-supplementary.pdf]

Rapid and Sensitive Fluorescent RT-RAA Assay for the Detection of a Panel of Six Respiratory Viruses

Xudong Guo [1,†], Dongli Gao [2,†], Yi Yang [1], Wanying Liu [1], Hongbo Liu [1,*], Rongtao Zhao [1,*] and Hongbin Song [1,*]

1 Chinese PLA Center for Disease Control and Prevention, Beijing 100071, China; placdcgxd@163.com (X.G.); flywolfg20251@163.com (Y.Y.)

2 Jiangyin Center for Disease Control and Prevention, Jiangyin 214400, China

* Correspondence: mailoflhb@126.com (H.L.); zhaorongtao1984@163.com (R.Z.); hongbinsong@263.net (H.S.)

† These authors contributed equally to this work.

Table S1. A comparison of major isothermal amplification methods with PCR highlights their distinct trade-offs.

Amplification methods	PCR	LAMP	NASBA	RPA/RAA
Template	DNA and RNA	DNA and RNA	RNA	DNA and RNA
Reaction temperature	Thermal Cycling Required (e.g., 94°C, 55–65°C, 72°C)	65°C	37-42°C	37-42°C
Amplification time	60-120 min	60 min	90 min	10-30 min
Number of primers	2	4-6	2	2
Detection methods for the amplified products	Real-time fluorescence, gel electrophoresis, etc.	Double-stranded chimeric dye, turbidimetry, indicator lateral flow chromatography, gel electrophoresis, and ELISA	Molecular beacon probes, gel electrophoresis, and ELISA	Gel electrophoresis and fluorescent probes
Advantages	Highest sensitivity & specificity, quantitative, gold standard	Simple reaction system, multiple detection methods, and product detection with naked eyes	Direct detection of RNAs and prevention of contamination	Rapid detection, low and constant temperature
Disadvantages	Relies on stable power, complex instruments, and lab setting	False positivity, complex primer design, and being prone to nonspecific amplification	A necessary preheating step and failure in achieving a real constant temperatur	Longer primer probes and being easy to form dimers

Table S2. Information on viral nucleic acid standards.

Name	Sequence Properties	Concentration (copies/mL)
SARS-CoV-2	N-gene，MT027064.1	5.54×10^{8}
HPIV1	Full-length genomic RNA	1.21×10^{5}
Flu A(H1N1)	segment7, NC_002016.1;	6.03×10^{7}
Flu B RNA standard（type Victoria）	segment5, CY018656.1	3.37×10^{8}
RSVA RNA standard	Full-length genomic RNA	1.68×10^{5}
ADV*（MW816018.1）	Full-length genomic DNA	1.34×10^{12}

*The adenovirus (ADV) sample was a concentrated viral stock from a laboratory archive, originally propagated in chicken embryo culture. Its genomic DNA concentration was measured via Nanodrop, and the copy number was calculated based on the full-length ADV genome (GenBank: MW816018.1; ~36 kb) using the formula: copies/μL = (DNA concentration [g/μL] × 6.022×10^{23}) / (genome length [bp] × 660 g/mol). This accounts for its higher reported concentration compared to the commercial RNA standards.

Table S3. List of instruments, reagents, and reference materials.

Product Name	Vendor / Brand	Catalog Number / Model	Country of Origin
Qitian RAA-B6100 mixer	Jiangsu Qitian Genetic Biotechnology Co., Lt.	RAA-B6100	China
Genchek fluorescent detector	Hangzhou Zhongce Bio-Sci&Tech Co. Ltd.	Genchek	China
CFX96 Touch real-time PCR system	Bio-Rad Laboratories, Inc.	CFX96	USA
RT-RAA kit	Hangzhou Zhongce Bio-Sci&Tech Co. Ltd.	S004ZC	China
RAA kit	Hangzhou Zhongce Bio-Sci&Tech Co. Ltd.	S002ZC	China
Detection Kit for Influenza A virus（PCR -Fluorescence probing）	Aodong Testing and Inspection Co., Ltd.	J001A	China
Detection Kit for influenza B virus（PCR -Fluorescence probing）	Aodong Testing and Inspection Co., Ltd.	J029A	China
Detection Kit for human adenovirus （PCR -Fluorescence probing）	Aodong Testing and Inspection Co., Ltd.	J105A	China
Detection Kit for human Parainfluenza virus type 1（PCR -Fluorescence probing）	Aodong Testing and Inspection Co., Ltd.	J107A	China
Detection Kit for respiratory syncytial virus（PCR -Fluorescence probing）	Aodong Testing and Inspection Co., Ltd.	J104P	China
Detection Kit for 2019-nCoV（PCR -Fluorescence probing）	Guangzhou Daan Genomics Co., Ltd.	--	China
SARS-CoV-2	Shanghai Institute of Measurement and Testing Technology	SNM0001	China
HPIV1	Shanghai Institute of Measurement and Testing Technology	SNM0004	China
Flu A(H1N1)	Shanghai Institute of Measurement and Testing Technology	SNM0006	China
Flu B RNA standard（type Victoria）	Shanghai Institute of Measurement and Testing Technology	SNM0007	China
RSVA RNA standard	Shanghai Institute of Measurement and Testing Technology	SNM0013	China

RSVB RNA standard	Shanghai Institute of Measurement and Testing Technology	SNM0014	China
MERS-CoV	Shanghai Institute of Measurement and Testing Technology	SNM0009	China
Rhinovirus types A	Shanghai Institute of Measurement and Testing Technology	SNM0010	China
Rhinovirus types B	Shanghai Institute of Measurement and Testing Technology	SNM0011	China
Rhinovirus types C	Shanghai Institute of Measurement and Testing Technology	SNM0012	China
H7N9	Shanghai Institute of Measurement and Testing Technology	SNM0005	China
HMPV RNA	Guangzhou Bondsheng Biotechnology Co., Ltd.	BDS-IQC-269	China
HCoV-OC43	Guangzhou Bondsheng Biotechnology Co., Ltd.	BDS-IQC-235	China
HCoV-229E	Guangzhou Bondsheng Biotechnology Co., Ltd.	BDS-IQC-241	China
HCoV-NL63	Guangzhou Bondsheng Biotechnology Co., Ltd.	BDS-IQC-244	China
Mycoplasma pneumoniae (MP) DNA	Guangzhou Bondsheng Biotechnology Co., Ltd.	BDS-IQC-008	China
SARS-CoV RNA	Guangzhou Bondsheng Biotechnology Co., Ltd.	BDS-IQC-247	China
Chlamydia pneumoniae (CP) CP DNA	Guangzhou Bondsheng Biotechnology Co., Ltd.	BDS-IQC-319	China
ADV	retrieved from our archived laboratory specimen repository		
Staphylococcus aureus (SA)	retrieved from our archived laboratory specimen repository		
Klebsiella pneumoniae (KP)	retrieved from our archived laboratory specimen repository		

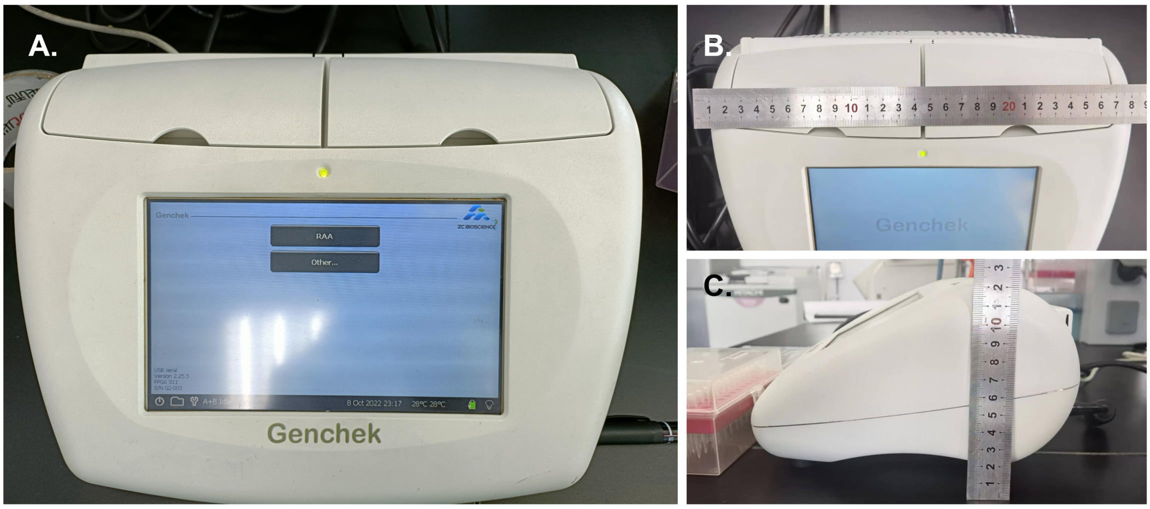

Figure S1. The portable Genchek real-time fluorescent RAA detector: photograph, physical dimensions, and key specifications. (A) Overview of the integrated Genchek detector unit, showcasing its compact and portable design suitable for field use. (B, C) Scale ruler measurements indicating the device's length (B) and height (C), providing direct physical reference.

Table S4. Key technical specifications of the portable Genchek real-time fluorescent RAA detector.

Specification[1]	Detail
Detection Method	Real-time fluorescent Recombinase-Aided Amplification (RAA)
Control & Data Management	Standalone operation via integrated touchscreen; supports on-board data storage, retrieval, and analysis.
Casing Material	PC/ABS alloy.
Dimensions	Length: 28 cm; Width: 20 cm; Height: 12 cm.
Weight	2 kg
Sample Throughput	16 reactions per run
Detection Channels	Dual channels (FAM, ROX)
Temperature Precision	≤ 0.5 °C
Built-in Battery	Rechargeable lithium battery (4800 mAh) supporting 4-5 hours of operation.
Charging Input	100-240 V AC.

[1] Specifications were obtained directly from the manufacturer.

Table S5. Identified conserved sequences of the six target viruses[1].

RSVA, (GenBank accession no. **KX655697.1**: 4610-5503)<br>ATGTCCAAAACCAAGGACCAACGCACCGCCAAGACACTAGAAAGGACCTGGGACACTCTCAA<br>TCATCTATTATTCATATCATCGTGCTTATACAAGTTAAATCTTAAATCTATAGCACAAATCACA<br>TTATCTATTTTGGCAATGATAATCTCAACCTCACTTATAATTGCAGCCATCATATTCATAGCCT<br>CGGCAAACCACAAAGTCACACTAACAACTGCAATCATACAAGATGCAACGAACCAGATCAAG<br>AACACAACCCCAACATACCTCACCCAGAATCCCCAGCTTGGAATCAGCTTCTCCAATCTGTCC<br>GGAACTACATCACAATCCACCACCATACTAGCTTCAACAACACCAAGTGCTGAGTCAACCCC<br>ACAATCCACAACAGTCAAGATCAAAAACACAACAACAACCCAAATACCACCCAGCAAACCCA<br>CCACAAAACAACGCCAAAATAAACCACAAAACAAACCCAACAATGATTTTCACTTTGAAGTG<br>TTCAATTTTGTACCCTGCAGCATATGCAGCAACAATCCAACCTGCTGGGCCATCTGCAAGAGA<br>ATACCAAACAAAAAACCTGGAAAGAAAACTACCACCAAGCCCACAAAAAAACCAACCATCA<br>AGACAACCAAAAAAGATCCCAAACCTCAAACCACAAAACCAAAGGAAGTACTCACCACCAA<br>GCCCACAGAAAAGCCAACCATCGACACCACCAAAACAAACATCAGAACTACACTGCTCACCT<br>CCAACACCACAGGAAATCCAGAACACACAAGTCAAGAGGAAACCCTTCACTCAACCACCTCC<br>GAAGGCAATCCAAGCCCATCACAAGTCTATACAACATCCG
HPIV, (GenBank accession no. **MF554715.1**: 82-1656)<br>ATGGCTGGGCTACTAAGTACTTTTGACACATTCAGCTCCAGGAGGAGTGAGAGCATCAATAA<br>GTCTGGCGGAGGAGCAATTATACCTGGTCAAAGAAGTACCGTTTCTGTCTTCACATTAGGCCC<br>GAGTGTGACAGATGATGCAGATAAATTATTAATAGCAACCACTTTCTTAGCCCACTCACTGGA<br>TACAGATAAACAACACTCTCAAAGAGGAGGATTTTTAGTATCACTCCTTGCAATGGCTTACAG<br>TAGCCCGGAGTTATATCTCACTACAAACGGTGTCAATGCTGATGTCAAGTATGTGATATACAG<br>TATAGAGAGAGATCCTAAAAGGACAAAAACAGATGGGTTCATTGTCAAAACAAGAGACATG<br>GAGTATGAAAGAACAACAGAGTGGTTGTTCGGACCTATGGTCAACAAGAACCCATTGTTCCA<br>GGGGCAAAGAGAGAATGCGGATCTAGAAGCATTGCTTCAGACATATGGATATCCTGCATGTC<br>TTGGAGCTATAATAGTTCAAGTTTGGATAGTGTTGGTTAAAGCCATAACAAGTAGTGCTGGTC<br>TAAGGAAAGGATTCTTCAATAGATTAGAAGCATTCAGACAGGATGGAACCGTTAAAAGTGCT<br>CTGGTCTTCACAGGAGACACAGTTGAAGGCATTGGTGCAGTGATGAGGTCACAACAAAGCTT<br>AGTATCTCTTATGGTAGAGACTCTAGTGACCATGAACACATCCAGGTCAGATCTAACTACACT<br>AGAGAAGAACATTCAGATTGTAGGAAATTACATAAGAGATGCAGGATTAGCATCTTTCATGA<br>ACACCATCAAGTATGGT

Flu A, (GenBank accession no. **NC_002018.1:** 1-1014)<br>AGCGAAAGCAGGGGTTTAAAATGAATCCAAATCAGAAAATAATAACCATTGGATCAATCTGT<br>CTGGTAGTCGGACTAATTAGCCTAATATTGCAAATAGGGAATATAATCTCAATATGGATTAGC<br>CATTCAATTCAAACTGGAAGTCAAAACCATACTGGAATATGCAACCAAAACATCATTACCTAT<br>AAAAATAGCACCTGGGTAAAGGACACAACTTCAGTGATATTAACCGGCAATTCATCTCTTTGT<br>CCCATCCGTGGGTGGGCTATATACAGCAAAGACAATAGCATAAGAATTGGTTCCAAAGGAGA<br>CGTTTTTGTCATAAGAGAGCCCTTTATTTCATGTTCTCACTTGGAATGCAGGACCTTTTTTCTG<br>ACCCAAGGTGCCTTACTGAATGACAGGCATTCAAATGGGACTGTTAAGGACAGAAGCCCTTA<br>TAGGGCCTTAATGAGCTGCCCTGTCGGTGAAGCTCCGTCCCCGTACAATTCAAGATTTGAATC<br>GGTTGCTTGGTCAGCAAGTGCATGTCATGATGGCATGGGCTGGCTAACAATCGGAATTTCAGG<br>TCCAGATAATGGAGCAGTGGCTGTATTAAAATACAACGGCATAATAACTGAAACCATAAAAA<br>GTTGGAGGAAGAAAATATTGAGGACACAAGAGTCTGAATGTGCCTGTGTAAATGGTTCATGT<br>TTTACTATAATGACTGATGGCCCGAGTGATGGGCTGGCCTCGTACAAAATTTTCAAGAT**CGAA**<br>**AAGGGGAA**GGTTACTAAATCAATAGAGTTGAATGCACCTAATTCTCACTATGAGGAATGTTC<br>CTGTTACCCTGATACCGGCAAAGTGATGTGTGTGCAGAGACAATTGGCATGGTTCGAACCG<br>GCCATGGGTGTCTTTCGATCAAAACCTGGATTATCAAATAGGATACATCTGCAGTGGGGTTTT<br>CGGTGACAACCCGCGTCCCAAAGATGGAACAGGCAGCTGTGGTCCAGTGTATGTTGATGGAG<br>CAAACGGAGTAA
Flu B, (GenBank accession no. CY018656.1:45-1727)<br>ATGTCCAACATGGATATTGACGGTATCAACACTGGGACAATTGACAAAACACCGGAAGAAAT<br>AGCTTCTGGAACCAGTGGGACAACCAGACCAATCATCAGACCAGCAACCCTTGCCCCACCAA<br>GCAACAAACGAACCCGTAACCCATCCCCAGAAAGAGCAACCACAAGCAATGAAGCTGATGTC<br>GGAAAGAAAACCCAAAAGAAACAGACCCCAACAGAGATAAAGAAGAGCGTCTACAACATGG<br>TAGTGAAACTGGGCGAATTCTATAACCAGATGATGGTCAAAGCTGGACTCAACGATGACATG<br>GAGAGAAACCTAATCCAAAATGCGCATGCTGTGGAAAGAATTCTATTGGCTGCCACTGATGA<br>CAAGAAAACTGAATTCCAGAAGAAAAAGAATGCCAGAGATGTCAAAGAAGGGAAAGAAGAA<br>ATAGATCACAACAAAACAGGAGGCACCTTTTACAAGATGGTAAGAGATGATAAAACCATCTA<br>CTTCAGCCCTATAAGAATTACCTTTCTAAAAGAAGAGGTGAAAACAATGTACAAAACCACCA<br>TGGGGAGTGATGGCTTCAGTGGACTAAATCACATAATGATTGGGCATTCACAGATGAATGAT<br>GTCTGTTTCCAAAGATCAAAGGCACTAAAAAGAGTTGGACTTGACCCTTCATTAATCAGTACC<br>TTTGCGGGAAGCACAATCCCCAGAAGATCAGGTGCAACTGGTGTTGCAATCAAAGGAGGTGG<br>AACTTTAGTGGCTGAAGCCATTCGATTTATAGGAAGAGCAATGGCAGACAGAGGGCTATTGA<br>GAGACATCAAAGCCAAGACTGCCTATGAAAAGATTCTTCTGAATCTAAAAAATAAATGCTCT<br>GCGCCCCAACAAAAGGCTCTAGTTGATCAAGTGATCGGAAGTAGAAATCCAGGGATTGCAGA<br>CATTGAAGATCTAACCCTGCTTGCTCGTAGTATGGTCGTTGTTAGGCCCTCTGTGGCGAGCAA<br>AGTGGTGCTTCCCATAAGCATTTACGCCAAAATACCTCAACTAGGGTTCAATGTTGAAGAGTA<br>CTCTATGGTTGGGTAC
SARS-CoV-2, (GenBank accession no. OQ253304.1: 28214-29464<br>TTCGTGGTGGTGACGGTAAAATGAAAGATCTCAGTCCAAGATGGTATTTCTACTACCTAGGAA<br>CTGGGCCAGAAGCTGGACTTCCCTATGGTGCTAACAAAGACGGCATCATATGGGTTGCAACT

GAGGGAGCCTTGAATACACCAAAAGATCACATTGGCACCCGCAATCCTGCTAACAATGCTGC<br>AATCGTGCTACAACTTCCTCAAGGAACAACATTGCCAAAAGGCTTCTACGCAGAAGGGAGCA<br>GAGGCGGCAGTCAAGCCTCTTCTCGTTCCTCATCACGTAGTCGCAACAGTTCAAGAAATTCAA<br>CTCCAGGCAGCAGTAGGGGAACTTCTCCTGCTAGAATGGCTGGCAATGGCGGTGATGCTGCTC<br>TTGCTTTGCTGCTGCTTGACAGATTGAACCAGCTTGAGAGCAAAATGTCTGGTAAAGGCCAAC<br>AACAACAAGGCCAAACTGTCACTAAGAAATCTGCTGCTGAGGCTTCTAAGAAGCCTCGGCAA<br>AAACGTACTGCCACTAAAGCATACAATGTAACACAAGCTTTCGGCAGACGTGGTCCAGAACA<br>AACCCAAGGAAATTTTGGGGACCAGGAACTAATCAGACAAGGAACTGATTACAAACATTGGC<br>CGCAAATTGCACAATTTGCCCCCAGCGCTTCAGCGTTCTTCGGAATGTCGCGCATTGGCATGG<br>AAGTCACACCTTCGGGAACGTGGTTGACCTACACAGGTGCCATCAAATTGGATGACAAAGAT<br>CCAAATTTCAAAGATCAAGTCATTTTGCTGAATAAGCATATTGACGCATACAAAACATTCCCA<br>CCAACAGAGCCTAAAAAGGACAAAAAGAAGAAGGCTGATGAAACTCAAGCCTTACCGCAGA<br>GACAGAAGAAACAGCAAACTGTGACTCTTCTTCCTGC
ADV, (GenBank accession no. MW816018.1:18385-21189)<br>TCAGGGGCTGGTCCTTCACCAGACTCAAAACCAAGGAGACTCCATCTCTTGGATCAGGGTTCG<br>ATCCCTACTTCGTTTATTCTGGATCTATTCCCTACCTGGATGGCACTTTTTACCTTAACCACACT<br>TTCAAGAAGGTCTCCATCATGTTTGACTCCTCAGTCAGCTGGCCTGGCAATGACAGGCTGTTG<br>TCTCCAAATGAGTTTGAAATCAAGCGCACTGTGGATGGGGAAGGATACAATGTGGCCCAATG<br>CAACATGACCAAAGACTGGTTCCTGGTTCAGATGCTTGCCAACTACAACATTGGCTACCAGGG<br>CTTTTACATCCCTGAGGGATACAAGGATCGCATGTACTCCTTTTTCAGAAACTTCCAGCCTATG<br>AGCAGGCAGGTGGTTGATGAGGTTAATTACACTGACTACAAAGCCGTCACCTTACCATATCAA<br>CACAACAACTCTGGCTTTGTAGGATACCTTGCGCCTACTATGAGACAAGGGGAACCTTACCCA<br>GCCAATTATCCATACCCGCTCATCGGAACTACTGCCGTTAAAAGTGTTACCCAAAAAAAGTTC<br>CTGTGCGACAGGACCATGTGGCGCATACCGTTCTCCAGCAACTTCATGTCCATGGGAGCCCTT<br>ACGGACCTGGGACAGAACCTGCTCTATGCCAACTCGGCCCATGCGCTGGACATGACTTTTGAG<br>GTGGATCCCATGGATGAGCCCACCCTGCTTTATCTTCTTTTCGAAGTCTTCGACGTGGTCAGAG<br>TGCACCAGCCACACCGCGGCGTCATCGAGGCCGTCTACCTGCGCACACCGTTCTCGGCCGGCA<br>ACGCCACCACATAA

[1]Primers and probes are denoted in blue and green, respectively, with their overlapping regions shown in yellow.

Table S6. Formulation of the RT-RAA fluorescence reaction mixture.

Reagent name	Volume（μL）
lyophilization tube	1tube
Buffer A	25
Forward primers	2
Reverse primers	2
probe	0.6
Buffer B	2.5
De-RNAase water	12.9
Template	5

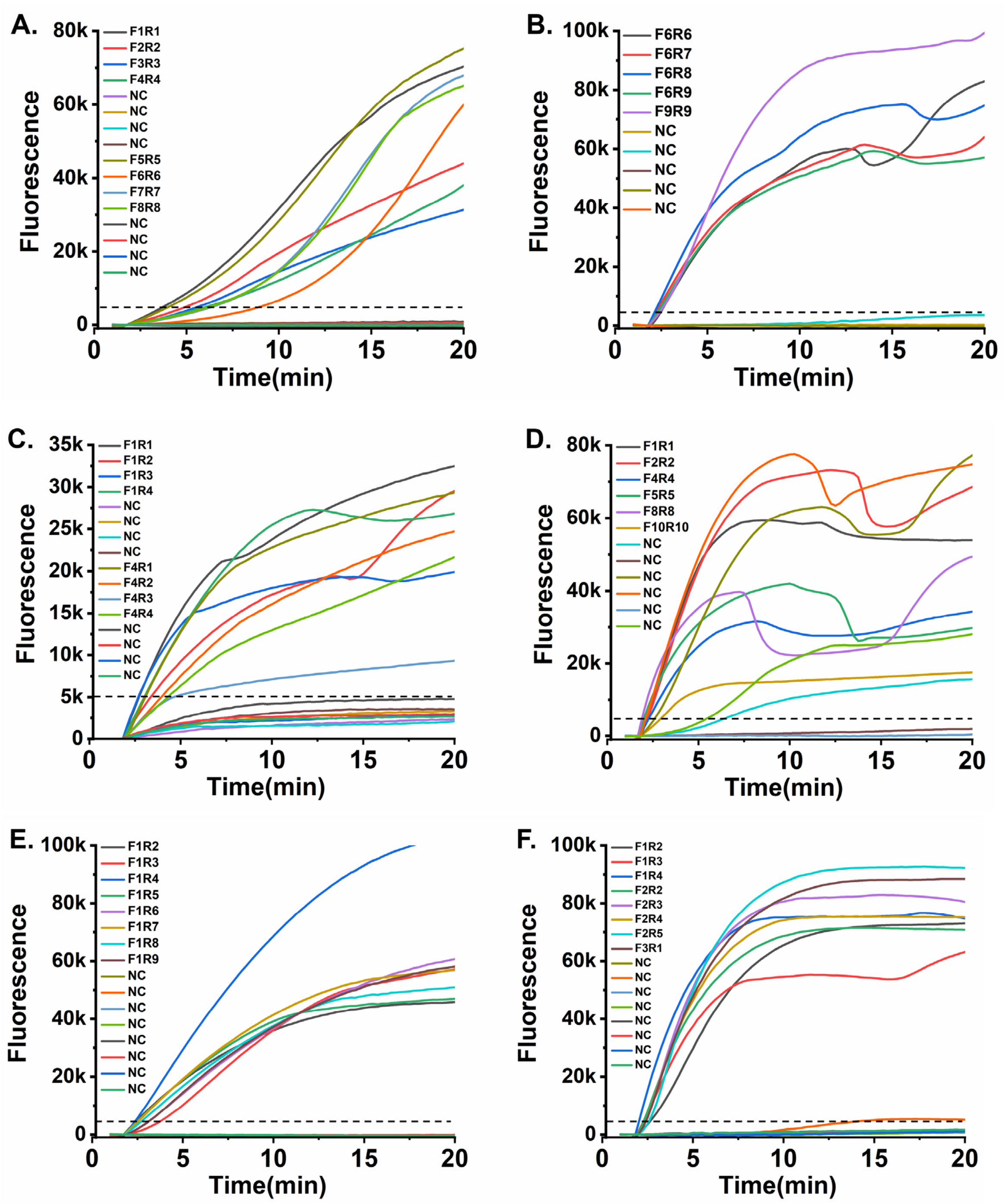

Figure S2. Evaluation of candidate primer-probe sets for the six respiratory viruses. Fluorescence-time curves from the screening process are shown for (A) RSV A, (B) HPIV, (C) Flu A, (D) Flu B, (E) SARS-CoV-2, and (F) ADV. The dashed horizontal line indicates the fluorescence threshold (5000 RFU).

Table S7. Detection results of RT-RAA versus RT-qPCR in clinical pharyngeal swab samples.

Assay	Virus		RT-PCR	
			Positive	Negative
RT-RAA	RSVA	Positive	11	0
		Negative	0	14
	HPIV	Positive	5	0
		Negative	0	14
	Flu A	Positive	6	0
		Negative	0	14
	Flu B	Positive	30	0
		Negative	0	14
	SARS-CoV-2	Positive	11	0
		Negative	0	14
	ADV	Positive	8	0
		Negative	0	14
